# A cross talk between microbial metabolites and host immunity: Its relevance for allergic diseases

**DOI:** 10.1002/clt2.12339

**Published:** 2024-02-11

**Authors:** Purevsuren Losol, Magdalena Wolska, Tomasz P. Wypych, Lu Yao, Liam O'Mahony, Milena Sokolowska

**Affiliations:** ^1^ Department of Internal Medicine Seoul National University Bundang Hospital Seongnam Korea; ^2^ Department of Molecular Biology and Genetics School of Biomedicine Mongolian National University of Medical Sciences Ulaanbaatar Mongolia; ^3^ Laboratory of Host‐Microbiota Interactions Nencki Institute of Experimental Biology Polish Academy of Sciences Warsaw Poland; ^4^ APC Microbiome Ireland University College Cork Cork Ireland; ^5^ Department of Medicine University College Cork Cork Ireland; ^6^ School of Microbiology University College Cork Cork Ireland; ^7^ Swiss Institute of Allergy and Asthma Research (SIAF) University of Zurich Davos Switzerland

**Keywords:** allergy, immune metabolism, immune response, microbial metabolites, microbiome

## Abstract

**Background:**

Allergic diseases, including respiratory and food allergies, as well as allergic skin conditions have surged in prevalence in recent decades. In allergic diseases, the gut microbiome is dysbiotic, with reduced diversity of beneficial bacteria and increased abundance of potential pathogens. Research findings suggest that the microbiome, which is highly influenced by environmental and dietary factors, plays a central role in the development, progression, and severity of allergic diseases. The microbiome generates metabolites, which can regulate many of the host’s cellular metabolic processes and host immune responses.

**Aims and Methods:**

Our goal is to provide a narrative and comprehensive literature review of the mechanisms through which microbial metabolites regulate host immune function and immune metabolism both in homeostasis and in the context of allergic diseases.

**Results and Discussion:**

We describe key microbial metabolites such as short‐chain fatty acids, amino acids, bile acids and polyamines, elucidating their mechanisms of action, cellular targets and their roles in regulating metabolism within innate and adaptive immune cells. Furthermore, we characterize the role of bacterial metabolites in the pathogenesis of allergic diseases including allergic asthma, atopic dermatitis and food allergy.

**Conclusion:**

Future research efforts should focus on investigating the physiological functions of microbiota‐derived metabolites to help develop new diagnostic and therapeutic interventions for allergic diseases.

## 1 INTRODUCTION

The human body hosts a vast array of microorganisms and their metabolites. The concentration of bacterial microbiota is highest in the colon (10^12^ bacteria/gram) and is dominated by anaerobic microorganisms belonging to the phyla Bacteroidetes, Firmicutes, and Proteobacteria.^1^, ^2^ The metabolic activity of the gut microbiome is essential in maintaining host homeostasis and health.^3^ The production of microbial bioactive metabolites is differentially influenced by a variety of factors, including environmental exposure, dietary and probiotic interventions, drugs, host genetics, and the alteration in an individual's metabolites that can all shape the immune system.^3^ The immunological functions of the metabolites generated from carbohydrates, tryptophan, bile acids, and polyamines have been extensively studied in the past decades.^4^, ^5^, ^6^ Their role in asthma and allergic diseases is a topic of growing interest.

Microbial diversity is highly variable between individuals. The gut microbiota is a dynamic ecosystem that changes over time, with lower diversity in early infancy and higher diversity in later childhood and adulthood.^7^ Short‐chain fatty acids (SCFAs), which are widely studied microbial metabolites, can regulate immune cell behavior through epigenetic modification of cell function and are involved in the prenatal programming of allergic diseases.^8^, ^9^ Maternal microbiome, birth mode, and feeding type are the early factors to impact compositional changes in the microbiota.^6^ Recent epidemiological studies suggest that living on a farm or exposure to a biodiverse environment is linked to microbiota diversity and protection against the development of allergic diseases.^10^, ^11^, ^12^

Patients with chronic inflammatory diseases, such as asthma and allergies, often have a reduced gut microbiota diversity.^13^, ^14^, ^15^, ^16^ Decreased microbial diversity and loss of SCFA‐producing bacteria are linked with greater epithelial damage and disease severity.^13^, ^14^, ^17^, ^18^ Asthma patients show an increased abundance of pathogenic *Moraxella*, *Streptococcus*, and *Haemophilus* in the airways, which are associated with eosinophilic inflammation and type 2 immune responses.^18^, ^19^ Atopic dermatitis‐related dysbiosis is characterized by the colonization of *Staphylococcus aureus* (*S. aureus*) and the simultaneous loss of other beneficial species in the skin.^20^ Metabolites propionate and butyrate from the abundant chronic rhinosinusitis bacteria *Fusobacterium nucleatum* alter the growth and gene expression of co‐colonizing *S. aureus*.^21^ In addition, gut microbiota‐derived tryptophan regulates immune cells in the lung, gut, and skin, ameliorating allergic airway inflammation and skin inflammation.^22^, ^23^, ^24^ Other microbial metabolites, such as bile acids, histamines, and polyamines, have also been documented to play a role in immune‐mediated allergic and non‐allergic diseases.^25^, ^26^, ^27^, ^28^ This review discusses the role of microbial‐derived metabolites in immune regulation and immune metabolism, and their association with diseases, with a particular focus on allergic and non‐allergic asthma, atopic dermatitis, and food allergy.

## 2 MICROBIAL METABOLITES

The gut microbiome can shape the immune system not only in the gut but also in distal body organs, such as the lungs, partly due to the secretion of immunomodulatory metabolites.^29^ Although the identity of most remains unexplored, there are a few metabolites active in the airways that have been described so far. The most common examples are SCFAs, bile acids, tryptophan metabolites, histamines, polyamines, and sphingolipids (Table 1).

**TABLE 1:** Functional role of microbial metabolites.

Metabolite		Cellular target/receptor	Mechanisms of action	Ref
SCFAs	Butyrate, acetate, propionate	Intracellular histone deacetylases (HDAC)	Inducing B cell differentiation and production of IgA and IgG via HDAC inhibition	88
			Suppressing FcεRI‐dependent cytokine release	183
			Enhancing FoxP3^+^ Treg cell differentiation via HDAC inhibition	184
		GPR41, GPR43, and GPR109a	Impairing the ability of DC to promote T helper 2 response, reducing ILC2 proliferation and function	85
			Enhancing tolerogenic CD103^+^ DC function via GPR43 and GPR109a, resulting in induction of oral tolerance to peanut antigens in mice	174
			Enhancing the generation of DCs with impaired ability to promote T helper 2 response via GPR41	36
Bile acids	UDCA	FXR	Promoting immunosuppressive phenotype in dendritic cells	57
	LCA	TGR5/GPBAR1	Inhibiting dendritic cell activation	185
Tryptophan	Kynurenine	Aryl hydrocarbon receptor (AHR)	Regulating IgE‐mediated responses in human mast cells (OVA challenge)	186
			Promoting Treg cell differentiation and attenuation of allergic responses	187
			Regulating dendritic cell immunogenicity	188
		TLR9‐induced IDO	Promoting IDO dependant immunosuppressive phenotype in pulmonary dendritic cells	23
	Indole derivatives	Aryl hydrocarbon receptor (AHR)	Promoting IL‐22 production by innate lymphoid cells	161
Polyamines	Spermidine	Unknown	Promoting immunosuppressive phenotype in dendritic cells	189
			Promoting CD4^+^ T‐cell differentiation towards regulatory phenotype Treg	69
			Reducing IL‐17 production in T cells	69
Tyrosine	p‐cresol sulfate (PCS)	TLR4‐EGFR interaction	Reducing CCL20 production in HDM‐stimulated airway epithelial cells	71
Histamine		H_2_R	Inducing IL‐10‐producing Treg cells and dendritic cells	50

Abbreviations: CCL20, C‐C motif chemokine ligand 20; DC, dendritic cells; EGFR, epidermal growth factor receptor; FcεRI, high‐affinity IgE receptor; FXR, farnesoid X receptor; GPBAR1 (TGR5), G‐protein‐coupled bile acid receptor; GPR, G protein‐coupled receptors; H_2_R histamine receptor 2; HDM, house dust mite; IDO, indoleamine 2,3‐dioxygenase; OVA, ovalbumin; TLR, Toll‐like receptor; Treg, T regulatory cells; UDCA, ursodeoxycholic acid.

### 2.1 SCFAs

**FIGURE 1:**
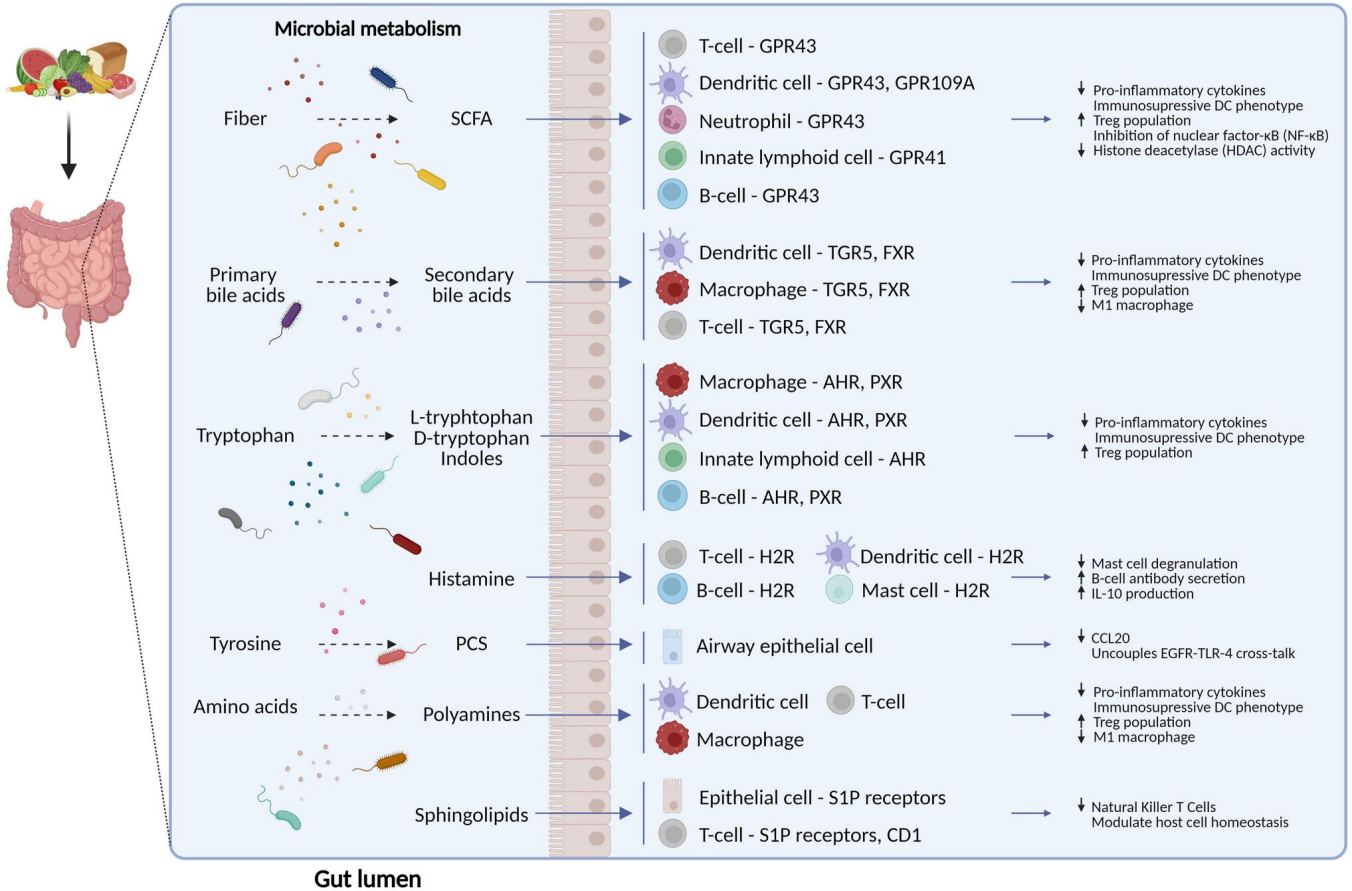
The interplay of gut microbiota‐derived metabolites and the immune system. Short‐chain fatty acids, derived from bacterial metabolism of dietary fibers, inhibit inflammation by binding membrane receptors (GPR41, GPR43, GPR109A) or inhibiting histone deacetylases (HDACs). Secondary bile acids, produced by bacterial transformation of primary bile acids, bind, among others, to membrane TGR5 (GPBAR1) or nuclear FXR receptors and inhibit inflammation. Tryptophan metabolites modulate the function of immune cells via aryl hydrocarbon receptor (AhR) and pregnane X receptor (PXR) receptors. Microbiota‐derived histamine modulates immune responses via histamine type 2 receptor (H2R). P‐cresol sulfate (PCS), derived from the microbial metabolism of L‐tyrosine, uncouples EGFR‐TLR‐4 cross‐talk and attenuates inflammation. Polyamines, generated by the metabolism of ingested amino acids, reduce pro‐inflammatory signaling through the receptors/pathways that are still to be determined. Microbiota‐derived sphingolipids can modulate immune responses via, among others, Sphingosine‐1‐phosphate receptors (S1PR) or by interacting with CD1d.

SCFAs are derived from gut microbial fermentation of dietary fibers (Figure 1).^30^ Most abundant SCFAs in the human gut are butyrate, for example, produced by *Faecalibacterium prausnitzii*, *Eubacterium hallii* and *Eubacterium rectale*; acetate, produced for example, by *Akkermansia muciniphila*, *Blautia hydrogenotrophica*, and *Bifidobacterium longum* ^31^; and propionate, produced for example, by *Roseburia inulinivorans*, *Bifidobacterium adolescentis*, and *Bacteroides fragilis*.^32^ Their mechanism of action often relies on the capacity to inhibit histone deacetylases (HDACs) and activate G‐protein‐coupled receptors (GPCRs).^33^ On the cellular level, this may lead to alterations of dendritic cell function and/or differentiation of Treg cells in the airways, as well as to the improvement of barrier integrity.^34^, ^35^, ^36^, ^37^

### 2.2 Tryptophan derivatives

L‐tryptophan is an essential amino acid that can be converted by the gut microbiome to indole and its derivatives, such as indole‐3‐acrylic acid (IA), indole acetic acid (IAA), indole‐3‐lactic acid (ILA), indole‐3‐propionic acid (IPA), and indole‐3‐carbaldehyde (I3C).^38^ Several bacteria were described to metabolize diet‐derived L‐tryptophan; *Escherichia coli* can convert dietary tryptophan to indole,^39^ *Clostridium sporogenes* produce tryptamine^40^ and Lactobacilli produce indole derivatives.^39^ Indole derivatives can modulate the immune response through the AHR receptor,^41^ influencing Th cell differentiation and cytokine synthesis towards an anti‐inflammatory state.^42^ Additionally, L‐tryptophan derivatives can directly modulate macrophage activation, while treatment with indole‐3‐butyric acid (IBA) and indole‐3‐lactic acid (ILA) was shown to suppress LPS‐induced cytokine production in vitro.^43^

### 2.3 Histamine

Histamine is generated by microbes following decarboxylation of the amino acid histidine, which can activate human histamine receptors similar to host‐derived histamine.^44^ High histamine levels are present in certain foods such as wine, tuna, mackerel, anchovy, spinach, sausage, dairy products, and fermented foods.^45^, ^46^ Of particular interest is the Histamine 2 Receptor (H2R), which can influence mast cell degranulation, the response of dendritic cells to microbial ligands, iNKT cell responses to lipid antigens, proliferation and cytokine production from T cells and antibody secretion by B‐cells.^47^, ^48^, ^49^, ^50^ An increased abundance of histamine‐secreting microbes, especially *Morganella morganii*, was observed within the gut of adult asthma patients, while histamine secretion from gut microbes could influence immune responses in the murine lung.^27^, ^51^

### 2.4 Bile acids

Bile acids (BA) are mainly synthesized in the liver, where they undergo conjugation to taurine or glycine, and are transported to the duodenum. A small amount that is not reabsorbed is enzymatically converted by the gut microbiome to secondary bile acids such as deoxycholic acid (DCA), lithocholic acid (LCA), and ursodeoxycholic acid (UDCA). Species associated with those conversions include *Clostridium scindens*, *C. hylemonae*, *Eggerthella lenta* (*Eubacterium lentum*), *Ruminococcus gnavus*, and *Bacteroides fragilis*.^52^, ^53^ Bile acids can directly interact with immune cells through specific receptors,^52^ including the nuclear farnesoid X receptor (FXR)^54^ and membrane G protein‐coupled bile acid receptor 1 (GPBAR1, also known as TGR5).^55^ GPBAR1 activation by taurolithocholic acid (TLCA) downregulated expression of pro‐inflammatory signaling in human macrophages, shifting classically activated macrophages (M1) to regulatory macrophage (M2b) phenotype.^56^ Ursodeoxycholic acid (UDCA) induced immunosuppressive phenotype on dendritic cells via nuclear FXR receptor.^57^ Bile acids can also modulate Treg cell populations. IsoalloLCA enhanced CD4^+^ T cell differentiation to Treg cells in vitro.^58^ Supplementation with either primary or secondary BAs increased colonic RORγ^+^ Treg cell counts and decreased colitis symptoms in mice.^59^

### 2.5 Sphingolipids

Sphingolipids are yet another group of microbial metabolites originating from both host and microbiome metabolism.^60^ The main dietary sources of sphingolipids are dairy products, eggs, soybeans, and fruits.^61^ In eukaryotic cells, they are structural membrane components that also function as signaling molecules.^62^ Out of gut commensals, *Bacteroides* is known to produce sphingolipids as part of its membrane.^63^ *Bacteroides fragilis*‐derived sphingolipid alpha‐galactosylceramide affected the numbers and function of natural killer T cells in a murine colitis model.^64^

### 2.6 Polyamines

Another microbiota‐derived immunomodulatory compound is polyamines, produced by transamination of ingested amino acids by gut microbiome, including *Bifidobacterium angulatum*, *B. animalis*, *B. faecale*, and *B. longum*.^64^, ^65^ The primary exposure to polyamines commences through breast milk and formula milk, subsequently to cereals, legumes, soy derivatives, as well as animal‐derived sources such as beef, pork, chicken, and sausages.^67^ Spermine and spermidine treatment decreased LPS‐induced production of pro‐inflammatory cytokines in macrophages.^68^ *In vitro*, spermidine induced differentiation of naive T cells into regulatory phenotype^69^ and activated indoleamine 2,3‐dioxygenase (IDO1)‐dependent immunosuppressive signaling in DCs.^39^, ^69^

### 2.7 P‐cresol sulfate

Finally, the immunomodulatory potential of L‐tyrosine metabolite, p‐cresol sulfate (PCS) has recently been demonstrated. PCS acted on airway epithelial cells to uncouple EGFR‐TLR‐4 cross‐talk, thereby attenuating CCL20 secretion in response to LPS or HDM.^71^

## 3 IMMUNE REGULATION BY MICROBIOME METABOLITES

### 3.1 Innate immune cells

Among the metabolites synthesized by the host or transformed by the microbiome, SCFAs, bile acids, and tryptophan catabolites are major signal molecules that are involved in intestinal epithelial integrity.^72^ SCFAs activate GPR41 and GPR43 on intestinal epithelial cells, inducing the production of cytokines (TNF‐α and IL‐6) and chemokines (CXCL1 and CXCL2), which promote the recruitment of neutrophils and activation of effector T cells.^73^ This leads to the induction of protective immune responses against pathogens. SCFAs promote the expression of antimicrobial molecules, RegIIIγ and *β*‐defensins, through GPR43, which are vital for the maintenance of intestinal homeostasis.^74^

Similarly, indole, a tryptophan metabolite, induces epithelial cell tight junction resistance, inhibits IL‐8 secretion and TNF‐α mediated NF‐κB activation, and reduces the expression of proinflammatory cytokines and chemokines.^75^ Indole and its derivatives exert biological functions through aryl hydrocarbon receptor (AhR), pregnane X receptor (PXR), and toll‐like receptor 4 (TLR4) expressed in various cells within the gut.^76^ The activation of AhR attenuates increased paracellular permeability of the intestinal epithelium and ameliorates localization of tight junction proteins.^77^ AhR deficiency enhances airway inflammation, mucus production, airway hyperresponsiveness, and remodeling in the chronic asthma model.^78^ Indole‐mediated activation of PXR is also an essential regulator of TLR4‐mediated control of the intestinal barrier function, and the deficiency of PXR decreases intestinal homeostasis and induces inflammation.^79^

Bile acids play an essential role in many aspects of the maintenance of intestinal barrier integrity.^80^ Alteration of the bile acid profile changes the intestinal permeability and affects the barrier function by regulating tight junction protein expression.^80^ High‐fat diet, which profoundly affects both the composition of the microbiota and of the bile acids pool, was shown to increase mucosal permeability by reducing the expression of occludin, antimicrobial peptide RegIIIβ, and IL‐22.^81^

### 3.2 Adaptive immune cells

SCFAs facilitate naïve T‐cell differentiation into the generation of Th1 and Th17 cells during active immune responses.^82^ Moreover, SCFA and its receptor‐signaling pathway induce the proliferation of Treg cells and upregulate the secretion of anti‐inflammatory cytokines, thereby suppressing allergic immune response.^83^ Innate lymphoid cells (ILCs) and CD4^+^ T cells produce IL‐22, which plays an important role in intestinal immunity. A study has shown that SCFA butyrate escalates the production of IL‐22 by CD4^+^ T cells and ILCs to maintain intestinal homeostasis.^84^ Butyrate also inhibits IL‐5 and IL‐13 production to modulate ILC2‐driven airway hyperresponsiveness and airway inflammation.^85^ SCFAs have been found to protect against food allergies by promoting immune tolerogenic mechanisms, including the maintenance of intestinal Treg cells and luminal IgA production.^83^, ^86^ Bile acid metabolites control Th17 and Treg cell differentiation in the intestinal lamina propria.^58^ Secondary bile acids have been reported to induce Foxp3 expression and RORγ^+^ regulatory T‐cell production in murine colitis model.^59^

In B cells, SCFAs are the major metabolites that regulate both mucosal and systemic antibody responses.^87^ SCFAs boost cellular metabolism and provide building blocks that support B cell activation, differentiation, and antibody production. SCFAs also control gene expression required for B‐cell differentiation.^87^ In allergic asthma, butyrate and propionate downregulate expression of activation‐induced cytidine deaminase (AID) and B lymphocyte‐induced maturation protein 1 (Blimp‐1), thereby reducing the rate of B‐cell class switching and the levels of circulating IgG1, IgA, and IgE.^88^ Patients suffering from rheumatoid arthritis were demonstrated to have lower levels of SCFAs, and the administration of these metabolites in vivo improved the symptoms of this condition.^89^

Tryptophan metabolites have both stimulatory and inhibitory effects on B cells. I3C can regulate B‐cell function by decreasing mature B cells and certain autoantibodies.^90^ Furthermore, IDO‐generated metabolites inhibit lipopolysaccharide‐induced antibody secretion and inhibit B‐cell apoptosis.^91^ IDO deficiency is associated with elevated levels of IgA that cross‐reacts with the enteric pathogen *Citrobacter rodentium*, demonstrating the role of IDO in the regulation of immunity to the gut microbiome.^91^

Finally, B cells contribute to maintaining bile acid homeostasis in the gut; consequently, impaired humoral immunity can disrupt this balance, leading to inflammatory gut diseases.^92^ Research suggests that the severity of intestinal inflammatory disease is influenced by both B cells and bile acids.^93^

## 4 MICROBIAL METABOLITES IN THE IMMUNE METABOLISM OF THE HOST

Immune metabolism is a term encompassing all intracellular biochemical processes leading to the proliferation, activation, and function of human immune cells.^94^ The focus of this developing research is to uncover how metabolic pathways govern and contribute to the regulation of appropriate immune responses within cells of the immune system, collectively known as immune metabolism. Certain metabolic programs support the immune functions of the cells, their differentiation, and phenotypic characteristics and therefore are increasingly recognized as cellular fingerprints, comparatively to cytokine or transcription factor profiles.^94^, ^95^ However, metabolism far exceeds the complexity of immunological phenotypes. Catabolic processes in the cell are responsible for energy production in the form of ATP. They include cytoplasmic anaerobic glycolysis and mitochondrial tricarboxylic acid (TCA), oxidative phosphorylation (OXPHOS), and fatty acid oxidation (FAO). Of those, only anaerobic glycolysis and OXPHOS create ATP directly, with OXPHOS being energetically much more efficient, but anaerobic glycolysis being much faster.^94^, ^95^ These pathways are interconnected with each other via common metabolites. For instance, glycolysis initiated in the cytoplasm after glucose transport creates not only ATP but also pyruvate, which is a substrate of TCA and thus participates in the TCA and OXPHOS, thus being called aerobic glycolysis. ATP in the cell is then used for anabolic needs such as nucleotide, protein, carbohydrates and lipid synthesis. Immune metabolism of the cell is thus finely regulated both intracellularly by expression of enzymes responsible for metabolic pathways, but also by availability of extracellular substrates for these processes, as well as by expression of membrane transporters.

Metabolism of bacteria also enables all of the processes needed for life and function of the bacterial cells and communities, often consisting of the same metabolites as in human cells.^96^ In addition, the human microbiome supplies unique enzymes and metabolites that aid in breaking down food in the gut and in performing xenobiotic metabolism.^96^ These microbial bioactive molecules encompass vitamins, amino acids, SCFAs, and other metabolites. These metabolites produced by microbiota, similar to host metabolites, may regulate many of the host cellular metabolic processes, exerting this way potent immunoregulatory functions,^96^, ^97^, ^98^, ^99^ presumably by modulating the metabolism of the host immune cells.

### 4.1 SCFAs

The most studied bacterial metabolites are SCFAs. They induce intestinal macrophages into more OXPHOS‐derived energy production^101^ and less glycolysis,^101^ resulting in enhancement of their antimicrobial activity and increased resistance to enteropathogens. Importantly, the broad disruption of the intestinal microbiome by antibiotic treatment increases the uptake of amino acids by colonic macrophages followed by the upregulation of the neutral amino acid transporter LAT1 (Slc7A5). After increased amino acids uptake, colonic macrophages increased both mTOR and related glycolysis, as well OXPHOS.^102^ SCFAs act as inhibitors of HDACs and thus modulate the epigenetic landscape, decreasing NF‐kB activation and the production of pro‐inflammatory cytokines by dendritic cells, monocytes and macrophages.^96^, ^102^, ^103^ Similarly, HDAC‐dependent regulation of FOXP3 expression in T regulatory cells have been extensively demonstrated in vitro and in vivo,^36^, ^104^ implicating profound metabolic reprogramming of Treg cells, but the exact metabolic pathways are yet to be determined. Moreover, SCFAs might induce direct changes in chromatin organisation by histone propionylation and butyrylation (H3K14pr and H3K14bu), again suggesting involvement of specific metabolic programmes.^106^ Similarly, SCFA recognition by GPR43, GPR41 and GPR109a, which are expressed by numerous cell types, including immune cells and intestinal epithelial cells,^96^, ^105^ induces the activation of several intracellular signaling pathways, but metabolic consequences are not yet known.

### 4.2 Tryptophan derivatives

Another group of metabolites being utilized and produced by intestinal microbiota are derivatives of tryptophan. Tryptophan is metabolised by the kynurenine, serotonin and indole pathways. *Clostridium*, *Bacteroides*, *Bifidobacterium* and *Lactobacillus* genera produce tryptophan metabolites.^106^, ^107^ They may act via AhR receptor, which is widely expressed in various cell types in the gut, lung and skin. Kynurenine metabolism, the major catabolic pathway for tryptophan, ends with the production of kynurenic acid and nicotinamide adenine dinucleotide (NAD^+^), both of which have broad effects on immune metabolism. Especially, NAD^+^ can act as an energy carrier and also as an enzymatic cofactor,^110^ directly influencing metabolic reprogramming. In addition, the intermediate metabolites of this pathway, such as hydroxykynurenine (3HK), 3‐hydroxyanthranilic acid (3HAA), and quinolinic acid (QA) have diverse effects on mitochondrial functions, influencing mitochondrial dynamics (fusion and fission events), mitochondrial related energy pathways (OXPHOS, TCA, FAO), mitochondrial DNA (mtDNA) stability and mitophagy.^111^ Main enzymes within the kynurenine pathways, such as indoleamine 2,3‐dioxygenase 1, 2 (IDO1,2) and tryptophan 2,3‐dioxygenase (TDO2) are expressed in immune cells such as macrophages,^112^ dendritic cells (DCs), B cells,^113^ and natural killer (NK) cells^114^ and T cells.^115^ Therefore, they may have profound effects on immune cell metabolism, although this has not been assessed systematically.

### 4.3 Amine oxides

The third group of microbial metabolites are Trimethylamine (TMA) and Trimethylamine N‐oxide (TMAO). Several metabolites of this group come from the degradation of ingested small molecules such as choline, L‐carnitine, or phosphatidylcholine, which are very common in nuts, meat, eggs, and dairy. TMAO, produced in hepatocytes, upon uptake of bacterial TMA and upregulation of enzyme FMO3, binds to the Protein Kinase RNA‐Like ER Kinase (PERK) and can induce the FoxO1 transcription factor in hepatocytes.^116^ TMAO, produced from TMA was demonstrated to be proatherogenic.^117^ Increased TMAO concentration impairs pyruvate and fatty acid oxidation in cardiac mitochondria,^118^ but not much is known about its influence of the metabolic reprogramming of immune cells.

### 4.4 Bile acids

Finally, bacteria can transform bile acids, creating a complex pool of secondary bile acids with many physiological functions.^119^ Interestingly, it was shown that one of the secondary bile acid 3β‐hydroxydeoxycholic acids (isoDCA) increased Foxp3 induction in Treg cells by acting on DCs via the FXR receptor,^120^ but again the exact pathway is not known.

## 5 ROLE OF MICROBIAL METABOLITES IN ALLERGIC DISEASES

### 5.1 Asthma

**FIGURE 2:**
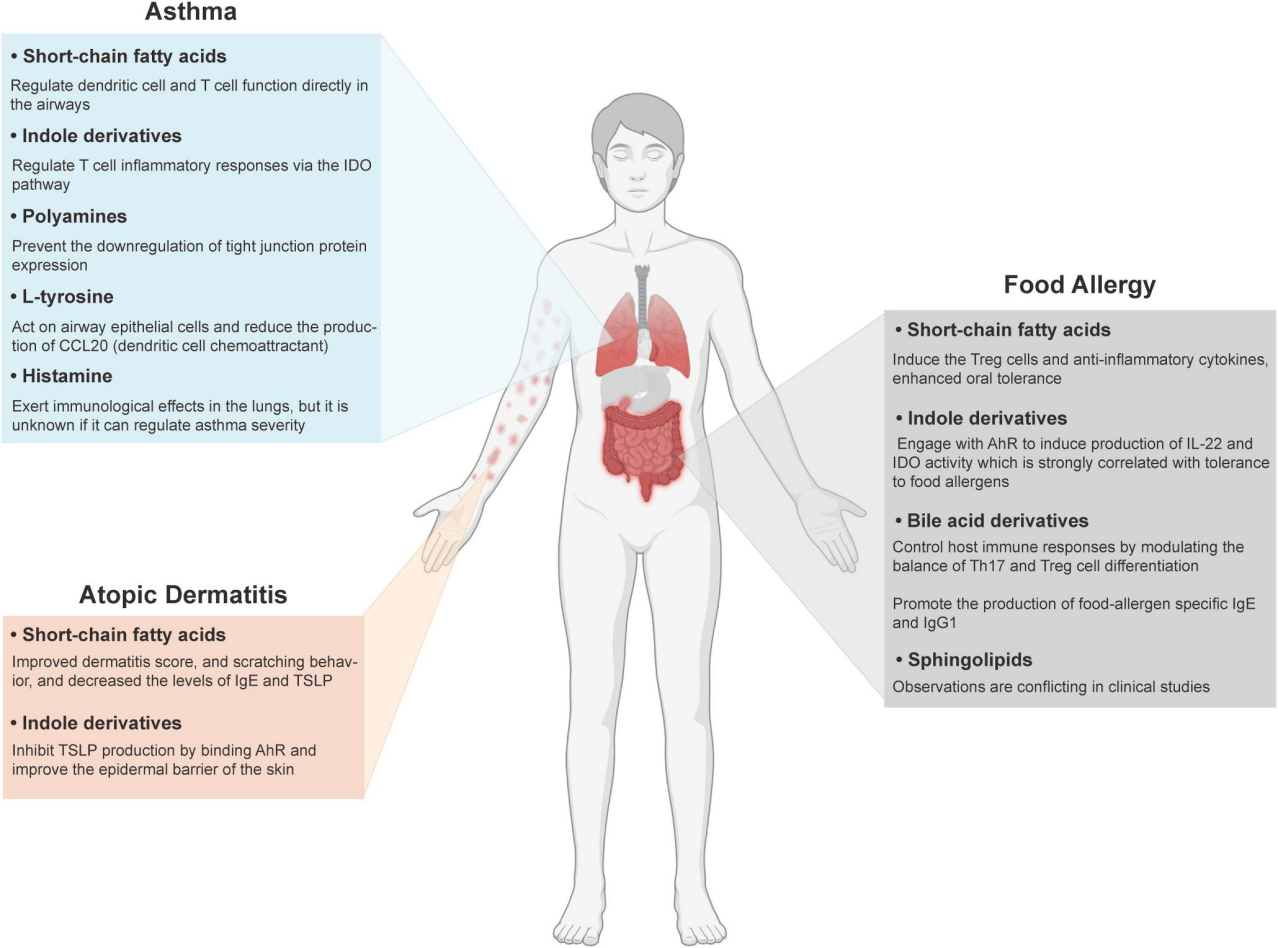
The function of microbial derived metabolites in different‐allergic diseases. In allergic asthma, short‐chain fatty acids, indole derivatives, polyamines, L‐tyrosine, and histamine have been proven to attenuate lung inflammation by regulating immune cell function and other mechanisms. In atopic dermatitis, short‐chain fatty acids and indole derivatives improve the disease severity through regulating the level of immunoglobulin E (IgE) and thymic stromal lymphopoietin (TSLP). In food allergy, short‐chain fatty acids, indole derivatives, and bile acid derivatives have shown to be connected to disease pathogenesis by affecting the lymphocyte differentiation and other pathways. The observation on the effects of sphingolipids remains conflicting in different clinical studies.

Asthma is a chronic respiratory disease affecting millions of people worldwide.^121^ There are multiple clinical phenotypes can be distinguished within asthmatic patients, with 2 endotypes having the highest incidence rate: Th2 low and Th2 high (usually observed in allergic and eosinophilic asthma) profiles. Allergic asthma is a heterogeneous disease characterized by excessive Th2 response of the adaptive immune system and consequent overproduction of proinflammatory cytokines IL‐4, IL‐5, and IL‐13.^122^ Dietary habits and the composition of the gut microbiota are well known to influence the severity of allergic asthma.^121^, ^122^ Aside from the gut microbiome, microbes locally inhabiting the airways may also affect asthma outcomes, since airway dysbiosis in asthmatic patients is well documented.^125^ The airways of asthmatic patients have an increased relative abundance of Proteobacteria (*Haemophilus*, *Neisseria*, and *Moraxella* genera),^126^ which might potentiate inflammation through the expression of immunogenic compounds, such as lipopolysaccharides or flagellin.^125^, ^126^ However, in the complete absence of microbes, asthma is also exacerbated as indicated by enhanced type 2 immunity of germ‐free mice, which can be restored by microbiota re‐colonization.^129^ These observations point to the importance of a balanced microbiota composition and microbial metabolites in maintaining lung homeostasis (Figure 2).

Though mechanisms that microbes employ to modulate immunity are numerous, their major mode of action involves the regulation of dendritic cell and T cell function directly in the airways. One way to achieve this is by secreting metabolites that reach distal body organs via circulation.

### 5.2 SCFAs

In the context of allergic asthma, prime examples are SCFAs, mainly butyrate, acetate, and propionate (Table 2). Butyrate and propionate levels in the feces of 1‐year‐old children were negatively correlated with the risk of developing asthma at 3–6 years of life.^130^ In animal models, a high‐fiber diet increased the levels of circulating SCFAs and alleviated the severity of allergic airway disease.^36^ Finally, supplementation with either butyrate, acetate, or propionate attenuated inflammation in a house dust mite model of murine asthma.^131^

**TABLE 2:** Clinical relevance of microbiota‐derived metabolites in allergic diseases.

Metabolite	Study design	Clinical implications and experimental findings	Ref
Allergic asthma and rhinitis			
SCFA	Experimental (mouse model)	High‐fiber diet increased circulating SCFA levels and protection against allergic inflammation in the lung.	36
		Oral administration of SCFA reduced the severity of allergic airway inflammation.	130
		Offspring from high‐fat diet mice had higher plasma SCFA, thymic, and peripheral Tregs.	190
		High‐fiber or acetate‐feeding suppressed allergic airway disease by enhancing Treg cells and expression of certain genes in the fetal lung linked to the disease.	123
	Experimental (mouse model), clinical trial & case‐control	Intestinal helminth infection reduced allergic asthma via alterations in the intestinal microbiota and the increase in SCFA production	191
		Fecal acetate levels were low in AW children. The concentration of butyrate was low in the AW mouse group.	25
	Case‐control	Reduced acetate and increased caproate were detected in the 3‐month fecal samples of children who developed atopic wheeze at age 5.	192
		Genes and pathways involved in the metabolism of SCFAs and amino acids were enriched in the asthmatic bronchial microbiome.	193
	Birth cohort	Children with the highest levels of butyrate and propionate in feces at the age of 1 yr had less atopic sensitization and less asthma development between 3 and 6 yrs. Children with the highest levels of butyrate had less FA or AR.	130
	Prospective cohort	Higher fecal acetic acid during pregnancy is associated with a lower risk of atopic asthma/wheeze in offspring at age 6.	194
Bile acids	Case‐control	The excretion of sulfated bile acids glycolithocholate, glycocholenate, and glycohyocholate was higher in AW children, whereas tauroursodeoxycholate excretion declined. In the urine of AW children, urobilinogen levels were increased 14‐fold.	25
		Asthma patients had higher plasma levels of taurocholate and glycodeoxycholate. Patients with high FeNO had higher levels of plasma branched‐chain amino acids and bile acids.	26
Tryptophan	Experimental (mouse model)	TLR9 ligand‐induced pulmonary IDO activity inhibited Th2‐driven asthma.	23
		D‐tryptophan administration increased lung and gut Tregs, decreased lung Th2 responses, and improved allergic airway inflammation and hyperresponsiveness.	22
	Case‐control	Patients with asthma and AR had higher neopterin, tryptophan, and kynurenine levels, and lower IgE levels and IDO‐1 enzyme activity.	195
		Allergic asthma children had lower IDO levels in the peripheral blood and sputum. IDO levels negatively correlated with FeNO.	196
	Prospective cohort	Pulmonary IDO activity was lower in patients with allergic asthma. Systemic tryptophan and its catabolites were higher in these patients. Systemic quinolinic acid and tryptophan were associated with ECP and eosinophils in BAL fluid and peak asthma symptom scores after the RV challenge.	134
Histamine	Case‐control	Patients with severe asthma had the highest levels of histamine‐secreting *M. morganii*.	27
Sphingolipids	Experimental (mouse model)	Sphingosine kinase inhibitor reduced inflammatory cell infiltrates, IL‐4, IL‐5, and eotaxin levels in BAL fluid, and suppressed airway hyperresponsiveness.	197
		SPP‐treated mice had increased mast cells and IL‐4, IL‐13, and IL‐17 production in the lung.	198
	Case‐control	Children with non‐allergic asthma had low levels of dihydroceramides, ceramides, and sphingomyelins. The results were inverse in children with allergic asthmatics.	199
		SPP levels were increased in asthmatics BAL fluid and modulated airway smooth muscle cell function. SPP inhibited TNF‐α‐induced RANTES release and induced IL‐6 secretion.	200
		Baseline lung function and severity of airway hyperreactivity correlated with plasma SPP level in HDM allergic patients. Allergen challenge increased plasma SPP concentration.	201
Atopic dermatitis			
SCFA	Case‐control & experimental (mouse model)	Propionate content was lower on the skin surface of AD patients. Topical application of propionate attenuated skin inflammation in mice by inhibiting IL‐33 production in keratinocytes and improved the symptoms of AD patients.	154
		AD patients had gut dysbiosis, dysregulated SCFA production, and increased IgE levels. Butyrate deficiency and downregulation of GPR109A and PPAR‐γ genes were observed in a mouse model of AD.	155
	Experimental (mouse model)	Oral butyrate strengthened skin barrier function, limiting allergen penetration, allergic sensitization, and disease development.	37
		Sodium butyrate (SB) application in sensitized mice reduced the contact hypersensitivity reaction. SB upregulated foxp3 and IL‐10 transcription.	202
	Experimental (human and mouse mast cell culture)	Propionate and butyrate inhibited IgE‐ and non‐IgE‐mediated mast cell degranulation.	203
	Case‐control	AD was characterized by gut dysbiosis and depletion of butyrate‐producing bacteria.	152
		The severity of eczema was associated with decreased butyrate‐producing bacteria and microbial diversity.	13
		AD patients had decreased fecal levels of butyrate and propionate.	153
	Birth cohort	Low fecal butyric acid was associated with an increased risk of developing wheezing, AD, and food sensitization till age 8 years.	157
		High levels of valeric acid at 3 years were associated with a low rate of eczema at the age of 8 years.	158
		Eczema at 13 years of age was negatively correlated with fecal valeric acid level at 1 year of age.	159
		Children with a lack of genes encoding enzymes for butyrate production develop allergic sensitization.	160
Tryptophan	Experimental (mouse model), case‐control & clinical trial	IDId levels were lower in the lesional and nonlesional skin of patients with AD. IAId attenuated skin inflammation in mice. IAId inhibited TSLP expression in keratinocytes.	24
		*Bifidobacterium longum* treatment upregulated tryptophan metabolism and increased fecal and serum indole‐3‐carbaldehyde to reduce AD symptoms.	162
	Cross‐sectional & experimental (cell culture)	Tryptophan metabolism was altered in allergic subjects. Indole‐3‐butyric acid (IBA) and indole‐3‐lactic acid (ILA) inhibited LPS‐induced upregulation of IL‐4 and IL‐6 in macrophages.	43
	Case‐control	AD patients had higher IDO‐1 activity and neopterin, tryptophan, and kynurenine levels.	195
Other metabolites	Case‐control	Microbe‐related methane and propanoate metabolisms were associated with AD. Metabolites dimethylamine and isopropanol were associated with host *FLG* mutations and serum IgE levels.	204
Food allergy			
SCFAs	Experimental (mouse model)	SCFAs induced the proliferation of Treg cells and upregulated the expression of anti‐inflammatory cytokines.	83
		High‐fiber diet protected against peanut allergy by reshaping the gut microbiome and increasing SCFA production.	174
	Case control	SCFAs were lower in FA patients and correlated with less abundant *Prevotella copri*.	172
		Extensively hydrolyzed casein formula with probiotic *Lactobacillus rhamnosus* (EHCF + LGG) promoted cow's milk allergy tolerance by influencing strain‐level bacterial community and increasing the levels of fecal butyrate.	176
	Birth cohort	Fatty acid metabolism was lower in the infant gut microbiome of children whose milk allergy resolved.	173
	Prospective cohort	EHCF + LGG treatment was associated with gut dysbiosis and butyrate production in non‐IgE‐mediated cow's milk allergy.	175
Bile acids	Experimental (mouse model)	Bile acid‐activated retinoic acid signaling promoted DC upregulation leading to Th2 differentiation of CD4^+^ T cells. Depletion of bile acid reduced food allergen‐specific IgE and IgG1 levels.	28
Tryptophan	Experimental (mouse model)	Mice with FAs had increased tryptophan metabolism with higher levels of indole derivatives.	177
		Fructooligosaccharides improved allergic symptoms and regulated Th17/Treg balance by modulating gut microbiome and tryptophan metabolism.	205
	Case‐control	History of severe systemic reactions and the presence of multiple FAs were associated with changes in tryptophan metabolites, eicosanoids, plasmalogens, and fatty acids.	180
		Indoleamine 2,3‐dioxygenase activity was lower in persistent childhood FA.	179
Sphingolipids	Prospective cohort	Sphinganine, 3‐ketosphinganine, 3‐hydroxypalmitate, N‐palmitoylserine, and 13‐methylmyristate were higher in subjects with food sensitization. Fecal iNKT‐cell activation was higher in subjects with food sensitization and was associated with sphingolipid metabolites.	181
	Case‐control	FA was associated with decreased sphingolipids.	180
		Children with FA had higher sphingolipid metabolites and lower acylcarnitine metabolites.	182

Abbreviations: Aire, higher autoimmune regulator expressed in the medulla of the thymus; AW, allergy skin prick test and clinical wheeze data at 1 year of age; BAL, bronchoalveolar lavage; ECP, eosinophil cationic protein; FA, food allergy; HDM, house dust mite; HYA, 10‐hydroxy‐cis‐12octadecenoic acid; IDId, Indole‐3‐aldehyde; IDO, indoleamine 2,3‐dioxygenase; RV, rhinovirus; SCFA, short‐chain fatty acid; SPP, sphingosine 1‐phosphate; TLR, toll‐like receptor; Treg, regulatory T cell.

### 5.3 Tryptophan derivatives

Tryptophan metabolism has been shown to regulate T cell inflammatory responses via the IDO pathway, by increasing tryptophan catabolism to kynurenine and shifting differentiation of naive T cells towards regulatory T cells.^130^, ^131^ IDO activity is also lowered in the blood of asthmatic children pointing towards the therapeutic potential of tryptophan metabolites and the signaling pathways they trigger.^134^ In a murine model, the administration of kynurenine‐induced IDO activity and attenuated OVA‐induced allergic asthma.^132^ Aside from microbial metabolism of L‐tryptophan, commensal bacteria can also secrete D‐tryptophan, which was shown to exert anti‐inflammatory properties. Oral administration of D‐tryptophan downregulated airway hyperresponsiveness and Th2‐associated immune responses in a murine model of allergic airway inflammation.^22^

### 5.4 Polyamines

Another class of metabolites, concentrations of which are associated with asthma outcomes, are polyamines. The levels of spermidine were shown to be decreased in the BAL but increased in the blood of asthmatic patients with active symptoms.^133^, ^134^ Mice receiving polyamines (spermidine or spermine) via oral gavage displayed reduced severity of HDM‐induced asthma and spermidine administration prevented HDM‐induced downregulation of tight junction protein expression.^136^

### 5.5 P‐cresol sulfate

Finally, a protective effect of L‐tyrosine metabolism was shown in the context of the HDM‐induced model of asthma. P‐cresol sulfate (PCS), the end‐product of microbial metabolism of L‐tyrosine, reached the airways through circulation, acted on airway epithelial cells, and reduced the production of a dendritic cell chemoattractant, CCL20. This resulted in impaired infiltration of dendritic cells into the lung tissue and attenuated type 2 inflammation.^71^

Collectively, the examples described above illustrate the potential of metabolites to influence immunity. In many cases, those metabolites can be produced by both host and microbial cells, and the exact contribution of metabolic pathways regulating each site of the ecosystem remains unknown. Likewise, administered metabolites might also induce tailored changes in the global metabolome profiles and thus, indirectly affect cellular processes. Regulatory networks within this interplay are complex and their deciphering will require efforts from multiple perspectives, ranging from metabolic, immunologic, and microbiologic standpoints.

Nonallergic asthma, which is not triggered by allergies, has been linked to Th1 and/or Th17 cell activation and steroid‐resistant neutrophilic inflammation.^137^ The compositions and metabolites of the gut microbiome are distinct between allergic and non‐allergic asthmatics. Metabolomics analysis revealed frequent changes in microbial metabolites and upregulation of histamine in non‐allergic asthmatics.^138^ Obese‐related asthma, a phenotype of nonallergic asthma, has been associated with an altered gut microbiota with a particular decrease in the abundance of *Akkermansia muciniphila*.^139^ Another nonallergic asthma that affects approximately 7% of asthmatic patients is aspirin‐exacerbated respiratory disease (AERD).^140^ Aspirin and/or NSAIDs can directly impact the composition and function of the gut microbiome or indirectly alter the physiological properties or functions of the host.^141^ NSAID‐induced changes in the microbiome promote the conversion of primary to secondary bile acids, resulting in more damage in the intestinal epithelial cells.^142^ On the other hand, without changing the bacterial population, celecoxib can decrease butyrate production in a human intestine and ameliorate inflammatory markers including IL‐8 and CXCL6 in intestinal cells, indicating the importance of the functional role of microbiome rather than taxonomic diversity.^143^

### 5.6 Atopic dermatitis

Atopic dermatitis (AD), also known as atopic eczema, is a chronic, inflammatory skin disease that involves genetic and environmental factors, and immunological responses. The prevalence is higher among young children, and 95% experience an onset below the age of 5.^142^, ^143^ Although AD resolves before adulthood, severe ADs with multiple factors such as early onset, filaggrin gene (*FLG*) mutations, and food allergies may persist into adulthood further developing other‐allergic co‐morbidities defined as the “atopic march”.^146^ AD patients have various systemic and skin immune abnormalities, including elevated serum IgE, sensitization to allergens, activated type 2 immune responses, and structural defect in skin‐barrier proteins.^145^, ^146^

Skin harbors heterogeneous microbial communities that are essential for barrier integrity, and skin and systemic immune homeostasis.^149^ Skin commensals *Staphylococcal epidermis* (*S. epidermidis*) and *Staphylococcus hominis* (*S. hominis*) produce antimicrobial peptides against pathogens and can decrease the abundance of *S. aureus*.^150^ Increased colonization of *S. aureus* in the skin of AD patients causes dysbiosis of the cutaneous microbiota and decreases bacterial diversity that is inversely correlated with AD severity.^14^, ^17^

### 5.7 SCFAs

The skin microbiome of AD patients has been shown to have a loss of anaerobic bacteria and a switch from anaerobic to aerobic metabolism.^148^, ^149^ Anaerobic bacteria in the skin ferment carbohydrates and amino acids, producing lactic acid and other SCFAs that lower the skin's pH.^151^ This acidic environment helps to protect the skin from infection and inflammation. A lower abundance of SCFA‐producing bacteria, predominantly *Bifidobacterium*, *Blautia*, *Coprococcus*, *Eubacterium*, and *Propionibacterium*, and decreased levels of SCFA metabolites are detected in the skin and intestinal samples of AD patients.^13^, ^150^, ^151^, ^152^, ^153^ A dominant gut bacterial species, *Faecalibacterium prausnitzii*, is a SCFA‐producer (in particular butyrate) and has been considered beneficial to gut health. The administration of these bacteria has improved dermatitis score, and scratching behavior, and decreased the levels of IgE and TSLP in vivo.^156^ However, enrichment of these species along with decreased levels of butyrate and propionate have also been reported in AD fecal samples.^153^ The loss of anaerobic bacteria in the skin microbiome of AD patients could explain the disturbed gut microbiome, leading to functional alterations of *F. prausnitzii* and dysregulation of gut epithelial inflammation in these patients. Individual birth cohort studies demonstrated that children having higher levels of SCFAs during infancy or at younger ages are less likely to develop allergic sensitization and AD.^155^, ^156^, ^157^, ^158^

### 5.8 Tryptophan derivatives

The skin of AD patients displays a lower level of tryptophan metabolites compared to that of healthy subjects.^24^ The skin microbiota‐derived Trp metabolite, indole‐3‐aldehyde (IAlD), can inhibit TSLP production in KCs by binding AhR and improving the epidermal barrier of the skin.^24^ Zelante et al. have shown that *Lactobacillus reuteri* produces an AhR ligand‐indole‐3‐acetaldehyde (I3A), followed by the induction of IL22 production, and balance mucosal reactivity.^161^ Similarly, Fang et al. demonstrated that probiotic *Bifidobacterium longum* upregulates tryptophan metabolism and increases fecal and serum I3A to reduce AD symptoms.^162^ These findings suggest that microbial‐derived metabolite IAlD has potent protection against AD.

AhR expressed in skin cells also acts as a sensor of environmental chemicals in the skin.^163^ AhR is activated by various external factors, including air pollutants, coal tar, and tryptophan metabolites that are generated by UV light exposure and/or by microbial tryptamine pathway.^159^, ^162^, ^163^ The activation of AhR via air pollution induces the expression of type 2 cytokines and the neurotrophic factor artemin, which promote AD symptoms.^166^ In addition, exposure of normal human epidermal keratinocytes to ozone increases the expression of cytochrome P450 isoforms through an AhR‐dependent mechanism,^167^ suggesting that the AhR pathway may be involved in coping with the air pollution‐mediated damage in the skin.

### 5.9 Food allergy

Food allergy (FA) is an adverse immune response to food proteins that can cause a range of symptoms from mild to severe. It is a growing problem in the world, affecting an estimated 1 in 10 people.^168^ IgE‐mediated FA disease differs from non‐IgE‐mediated FA in its pathophysiology. IgE‐mediated FA is caused by the activation of the immune system, which results in a Th2 response and the binding of IgE to Fcε receptors on effector cells. This triggers the release of histamine and other inflammatory mediators by mast cells and basophils, leading to the rapid onset of symptoms. In contrast, non‐IgE‐mediated FA has a delayed onset and is characterized by subacute or chronic inflammatory processes in the gut.^169^

### 5.10 SCFAs

The gut microbiome may be involved in the development of food allergies by influencing the production of Treg cells, which play a role in suppressing the immune response to food allergens.^170^ Promoting food tolerance by the induction of Treg cells can be achieved through microbial production of SCFAs.^171^ In observational studies, differences in the gut microbiota composition and microbial metabolites have been found between participants with FAs and healthy subjects, indicating that different microbiome may present distinct effects on oral tolerance.^170^, ^171^, ^172^, ^173^, ^174^ Patients with FA has less abundant commensal bacteria and lower levels of SCFAs. This loss of SCFAs can impair the function of the immune system and lead to a reduced ability to develop oral tolerance. In an experimental study, SCFA stimulation and a high‐fiber‐diet induced the expression of Treg cells and anti‐inflammatory cytokines, reshaped the gut microbiome, and enhanced oral tolerance.^82^, ^172^ Several reports investigating the potential benefits of probiotics in FA have been published so far. For example, the formula supplemented with *Lactobacillus rhamnosus* GG influences the bacterial community composition and butyrate production in infants with FA.^173^, ^174^

### 5.11 Tryptophan derivatives

A growing body of evidence suggests an association between FA and tryptophan metabolites. A study using a murine model has shown an important role of the intestinal microbiome in allergic responses through tryptophan metabolism and the production of indole derivatives.^177^ These derivatives engage with AhR to induce production of IL‐22.^178^ Of note, IDO activity, as assessed by kynurenine/tryptophan ratio is strongly correlated with tolerance to food allergens despite allergen sensitization.^179^

### 5.12 Bile acids

Distinct derivatives of BAs and sphingolipids have also been linked to FA pathogenesis.^28^, ^178^, ^179^, ^180^ BA derivatives are implicated in the regulation of gut microbiome and modulation of colonic RORγ^+^ Treg cell homeostasis.^59^ Derivatives of lithocholic acid (LCA), 3‐oxoLCA, and isoalloCLA, are involved in controlling host immune responses by modulating the balance of Th17 and Treg cell differentiation.^58^ In an animal study, food sensitization was mediated by bile acid‐activated retinoic acid signaling in DCs, which then promoted the production of food allergen‐specific IgE and IgG1.^28^

### 5.13 Sphingolipids

Finally, the association between sphingolipids and FA has been documented in both clinical and experimental studies.^178^, ^179^, ^180^ For example, a recent report identified low levels of serum sphingolipids and ceramids in patients with FA^180^, which is consistent with the protective effect of *Bacteroides*‐derived sphingolipids in sensitized individuals.^181^ However, certain observations contradict the beneficial role of sphingolipid metabolites in FA because they were also detected in higher concentrations in the serum samples of FA patients.^182^ Together, these observations indicate that the role of sphingolipids in FA may be more complex.

## 6 CONCLUSIONS

Increased diet diversity and environmental biodiversity may alter the taxonomic composition and metabolites of the gut microbiome, potentially increasing the risk of allergies. The impact of microbiota‐derived metabolites on the pathogenesis of immune‐mediated disease pathogenesis may be greater than previously appreciated. Metabolites synthesized by the host or generated by the microbiome contribute to intestinal barrier integrity, immune metabolism, tolerance, activation of immune effector cells, and expression of proinflammatory cytokines and antimicrobial molecules. This review brings together the current literature on microbial metabolites in allergic diseases, identifying mechanisms triggered by microbial metabolites that regulate host immunity and cellular metabolic processes. Future research will be key to identifying other microbiota‐derived metabolites' importance in these processes and understanding their interactions with other cells in health and disease. These insights might be important to identify new therapeutic targets to ameliorate inflammatory conditions, such as allergies and asthma.

## AUTHOR CONTRIBUTIONS

**Purevsuren Losol**: Conceptualization (equal); Writing – original draft (equal); Writing – review & editing (equal). **Magdalena Wolska**: Writing – original draft (equal); Writing – review & editing (equal); Preparing figure (equal). **Tomasz P. Wypych**: Conceptualization (equal); Writing – review & editing (equal). **Lu Yao**: Preparing figure (equal); Writing – review & editing (equal). **Liam O'Mahony**: Conceptualization (equal); Writing – review & editing (equal). **Milena Sokolowska**: Conceptualization (equal); Writing – original draft (equal); Writing – review & editing (equal).

## CONFLICT OF INTEREST STATEMENT

None.

## Data Availability

Data sharing not applicable to this article as no datasets were generated or analysed during the current study.
